# Variation in the Distribution of *Nosema* Species in Honeybees (*Apis mellifera* Linnaeus) between the Neighboring Countries Estonia and Latvia

**DOI:** 10.3390/vetsci8040058

**Published:** 2021-04-01

**Authors:** Sigmar Naudi, Juris Šteiselis, Margret Jürison, Risto Raimets, Lea Tummeleht, Kristi Praakle, Arvi Raie, Reet Karise

**Affiliations:** 1 Chair of Plant Health, Institute of Agricultural and Environmental Sciences, Estonian University of Life Sciences, Friedrich Reinhold Kreutzwaldi 1a, 51014 Tartu, Estonia; margret.jyrison@emu.ee (M.J.); risto.raimets@emu.ee (R.R.); reet.karise@emu.ee (R.K.); 2 Latvian Beekeepers Association, Rīgas iela 22, LV-3004 Jelgava, Latvia; juris.steiselis@strops.lv; 3 Institute of Veterinary Medicine & Animal Sciences, Estonian University of Life Sciences, Kreutzwaldi 62, 51006 Tartu, Estonia; lea.tummeleht@emu.ee (L.T.); kristi.praakle@emu.ee (K.P.); 4 Estonian Ministry of Rural Affairs, Lai tn 39, 15056 Tallinn, Estonia; arvi.raie@agri.ee

**Keywords:** *Apis mellifera* L., *Nosema ceranae*, *Nosema apis*, unicellular, pathogens

## Abstract

The unicellular spore-forming parasites *Nosema apis* and *Nosema ceranae* are considered to be one of the causes of increased honey bee mortality in recent years. These pathogens attack their honey bee hosts through their gut, causing changes in behavioral stress responses and possibly resulting in decreased honey yield and increased honey bee mortality. The present study aimed to determine the prevalence of *Nosema* spp. (nosemosis) in Estonia and Latvia, as well as the persistence of the disease in previously infected hives. Currently, *N. ceranae* is considered the most virulent species and is predominant worldwide. However, in some regions, usually with colder climates, *N. apis* is still prevalent. To achieve better disease control, it is important to determine the species distribution. For this purpose, we selected 30 apiaries in Estonia and 60 in Latvia that were positive for *Nosema* spp. in the EPILOBEE (2012–2014) study, which was 5 years prior to the present study. The results show that, while both species are present in Estonia and Latvia, *N. apis* is dominant in Estonia (43%), and *N. ceranae* is dominant in Latvia (47%). We also found that the pathogens are very persistent, since 5 years later, only 33% of infected apiaries in Estonia and 20% of infected apiaries in Latvia, we could not detect any pathogens at the time of sampling.

## 1. Introduction

The biggest challenge for beekeepers, from both an economic and ecological perspective, is limiting winter mortality, which means that the honey bee colonies must be healthy. In recent years, winter mortality has been relatively high, which has led to collaboration between scientists and beekeepers to determine the causes [1]. Proposed explanations include increased environmental pressures due to changes in land use [2], the presence of pesticide residues in nectar and pollen [3,4], and changes in the distribution of parasites and pathogens [5]. In northern countries such as Estonia, temperatures have increased and winters are milder, and in the last 10 years, scientists have observed temperature-related changes in insect populations. For example, the typically univoltine Colorado potato beetle can now produce two viable generations in warmer years [6].

Chronic exposure to stressors can cause disorders of the honey bee immune system [7]. One of these stressors is acute *Nosema* infection, which often causes no symptoms, but can decrease honey bee immunity [8], which in turn increases the risk of mortality. Nosemosis is a honeybee disease caused by the unicellular spore-forming fungal parasites *Nosema apis* Zander [9] and *N. ceranae* Fries et al. [10] (division *Microsporidia*) [11]. *N. apis* was thought to be the only cause of nosemosis in the honey bee *Apis mellifera* L. until 2005, when *N. ceranae* was first described in *A. mellifera* L. in Taiwan [12]. The first *N. ceranae* infection was also found in Spain at the same time [13].

*N. ceranae* has replaced *N. apis* in many countries [14,15]; it has become predominant in Argentina [16], central Italy [17], Croatia [18], Serbia [19], and South-Germany [20]. *N. ceranae* is also dominant in more northern countries, such as Lithuania and Finland [21,22]. Essentially, *N. ceranae* has spread throughout the world in a short time.

Nosemosis damages the tissues in the honey bee midgut, is energy-consuming for the host, causes changes in behavioral stress responses, and may reduce the life span of the host [23]. Both *N. ceranae* and *N. apis* affect the epithelial cells of the gut, but it has long been thought that *N. ceranae* can also exist in the hypopharyngeal glands, salivary glands, Malpighian tubules, and body fat [24]. However, recently, it was shown that *N. ceranae* has a specific tropism for the epithelial gut, as this was only tissue invaded by this parasite [25]. Despite their generally similar descriptions, these two species affect honey bees differently. *N. apis* outbreaks occur mostly during the springtime and tend to be less severe, while *N. ceranae* outbreaks are detected during the entire period of active colony growth and can cause gradual fading [26]. The hidden course of the disease is also one of the reasons why it is difficult to eliminate. Furthermore, in Europe, no veterinary drugs are registered to control it, because these fungi have developed resistance to antibiotics, which are now ineffective and can leave toxic residues in the hive. The antibiotic fumagillin has been used in beekeeping and has been shown to be effective against nosemosis. However, fumagillin is fairly toxic, can cause chromosomal aberrations, and is carcinogenic to human consumers of honey bee products [27]. Thymol has shown good results in laboratory studies [28,29], and good results against *Nosema* have also been shown for various nutraceutical and immunostimulatory compounds. However, further research is needed in this area [30]. European beekeepers use different measures to control *Nosema* infection. Most of these are hygienic management techniques, but selecting and replacing infected colonies and changing the queen may also help mitigate the infection [31].

The symptoms of nosemosis can vary and can be inconspicuous. The two *Nosema* species are distinguished according to their clinical pattern: *N. apis* causes nosemosis type A, and *N. ceranae* causes nosemosis type C [32]. The latter is more problematic because there is no specific clinical picture. However, the disease might lead to a decrease in honey production and may contribute to mortality [33].

The European EPILOBEE project, which was conducted in 2012–2014, mapped the spread of honey bee viruses and parasites in the member states of the European Union [1]. During the project, 197 samples were taken from hives with clinical symptoms of nosemosis in Estonia in 2012, and 30 of these were positive, and 194 samples were collected from hives in Latvia in 2013, and 60 of these were positive. The persistent nature of the disease, difficulties in self-diagnosis, and variation in the clinical symptoms of the disease caused by the two different species resulted in the need to repeat the survey. Therefore, we aimed to resample previously *Nosema*-positive apiaries to assess the persistence of the disease and determine the distribution of *N. apis* and *N. ceranae* in Estonia and Latvia.

## 2. Materials and Methods

### 2.1. The Geographical Location of Apiaries and Honey Bee Sampling

This study was undertaken in Estonia (2017) and Latvia (2018). Apiaries that had previously tested positive for nosemosis (EPILOBEE, 2012–2014), including 30 apiaries (1 apiary = 1 sample) in Estonia and 60 apiaries in Latvia, were resampled. Samples were collected before the major turnover from winter to summer bees (May) to obtain the largest number of spores per bee. In the sampling years, the spring was rather cold, and colonies were just starting their development. For each sample, 60 forage bees were collected from the flying boards of 2–4 neighboring hives using a portable vacuum device. The samples were placed in plastic tubes, cooled immediately for transportation, and frozen at −20 °C until laboratory analyses.

### 2.2. DNA Extraction and Analysis

Twenty worker honey bees were randomly selected from each sample, and their abdomens were removed using a sterile disposable scalpel. The abdomens were pooled and placed in a 15 × 28.5 cm Universal Extraction Bag (Bioreba). Then, 3 mL of ddH_2_O was added to facilitate homogenization using a hand homogenizer. Approximately 800 μL of the suspension was collected for DNA extraction.

DNA was isolated using the DNeasy Blood and Tissue Kit (Qiagen, Hilden, Germany) according to the manufacturer’s protocol. A multiplex PCR (M-PCR) assay was performed to identify the *Nosema* species using 2 μL of DNA and primers as described by Fries et al. (2013) [26] (Table 1). The PCR program consisted of a 2-min initial denaturation at 95 °C, followed by 35 cycles of denaturation for 30 s at 95 °C, annealing for 30 s at 57 °C, and elongation for 1 min at 72 °C, with a final elongation step at 72 °C for 5 min. The M-PCR products were visualized on a 2% agarose gel.

### 2.3. Flow Cytometry and Spore Counting

A flow cytometer (BD Accuri C6) was used to determine the number of spores per honey bee, and, in cases of a mixed infection, the total number of spores are presented. An aliquot (50 μL) of the suspension generated for DNA isolation was diluted with 1 mL of ddH_2_O, and 50 μL of the dilution was used for the spore counting analysis. A disposable sieve (10 µm) was used to remove debris. To determine the number of spores per honey bee, the following formula was used: $x=\frac{3000\;\times \;1050}{20\;\times \;50\;\times \;50}$ × SA (spores in the sample). The formula is explained as follows: 3000 (3000 μL of water added to facilitate homogenization), 1050 (final mixture from which 50 μL of spore suspension was removed was diluted with 1000 μL of water), 20 (number of bee abdomens used), 50 (50 μL of spore suspension used in the dilution), and 50 (50 μL of the filtered spore suspension was loaded into the flow cytometer for spore counting). The accuracy of the results was compared to the microscopic spore counts from five samples of each infected species. There was no significant difference between the results [KW-H = 0.06, *p* = 0.73].

### 2.4. Statistics

Data were processed using TIBCO Statistica^®^ 13.3.0. The Kruskal-Wallis test was used to assess the statistical differences in spore number per honey bee in Estonia and Latvia and confirm the accuracy of the flow cytometry results. The chi-square test, which compares the differences in shares of subdivisions, was used to examine the statistical significance of differences between *N. ceranae* and *N. apis* distribution changes in Latvia. Statistical significance was set at *p* < 0.05.

## 3. Results

### 3.1. Prevalence of N. apis and N. ceranae

**Figure 1:**
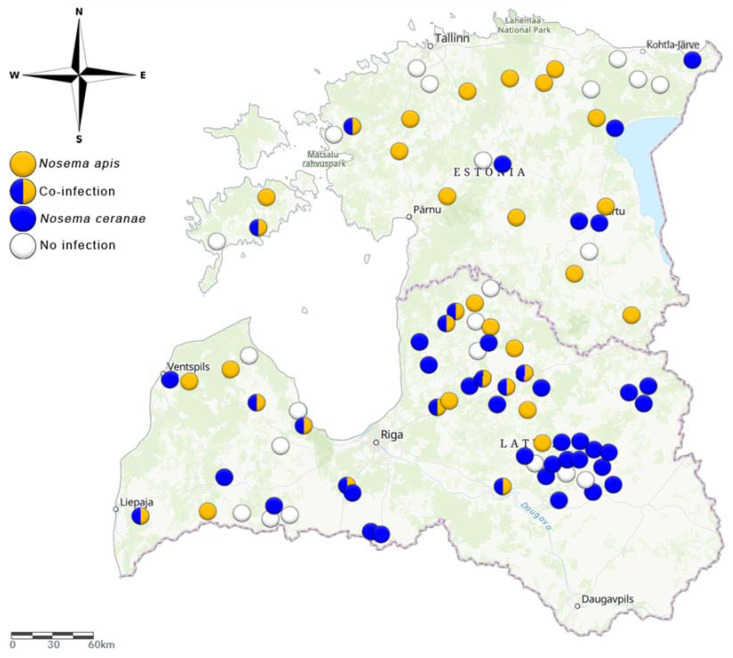
Prevalence of nosemosis causative agents (*Nosema ceranae* and *Nosema apis*) in Estonia in 2017 (May) and Latvia in 2018 (May) (ArcGis online).

Multiplex PCR showed that both Nosema species were present in Estonia and Latvia, either separately or in co-infections. The species distribution varied between the two countries. *N. apis* was the more prevalent species in Estonia, while *N. ceranae* was more prevalent in Latvia at the time of sampling. Among the 30 sampled apiaries in Estonia, 17% were positive for *N. ceranae*, 43% were positive for *N. apis*, and 7% were co-infected. In the remaining 33% of sampled apiaries, *Nosema* was no longer detected. In Latvia (n = 60), the results were almost the opposite: 47% of the samples were positive for *N. ceranae*, 15% were positive for *N. apis*, and 18% were co-infected. In the remaining 20% of sampled apiaries, *Nosema* was no longer detected in the sampling period (Figure 1).

### 3.2. Spore Quantity

**Figure 2:**
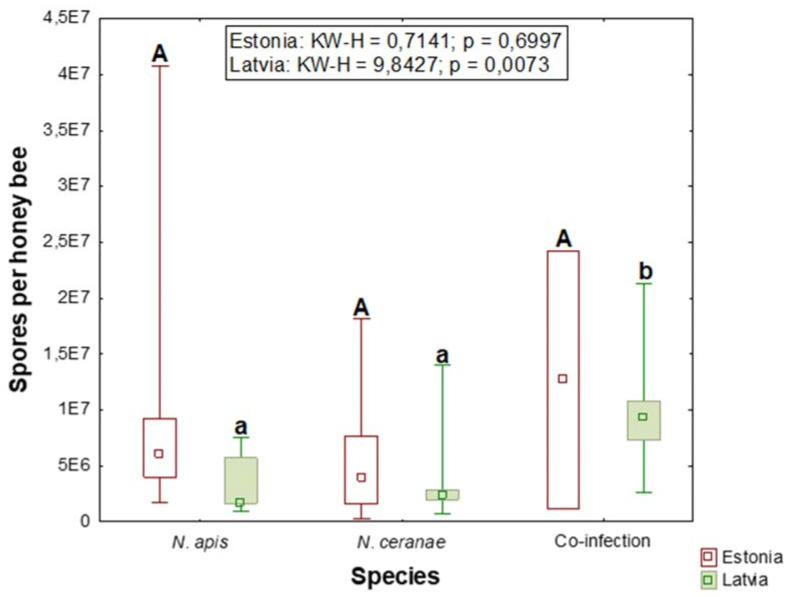
Bar graphs depicting the numbers of *Nosema* spores per honey bee in samples from Estonian and Latvian apiaries, as measured using flow cytometry. Error bars are the minimums and maximums. Columns with different letters indicate significant differences. Statistical differences were calculated by the Kruskal-Wallis test, with significance set at *p* < 0.05.

The number of spores per honey bee was the highest when both species were present. The median count (spores per honey bee) in co-infections was approximately 12 million in Estonia and approximately 9 million in Latvia. In single species infections, the median spore count for *N. apis* was approximately 6 million, while that for *N. ceranae* was approximately 4 million in Estonia. There were no statistically significant differences in spore counts between *Nosema* species or mixed infections (Figure 2). In Latvia, the median spore count for *N. apis* was approximately 1.7 million and that for *N. ceranae* was approximately 2.4 million spores per honey bee (Figure 2), which was significantly lower than that in co-infections.

### 3.3. Species Distribution Changes in Latvia

**Figure 3:**
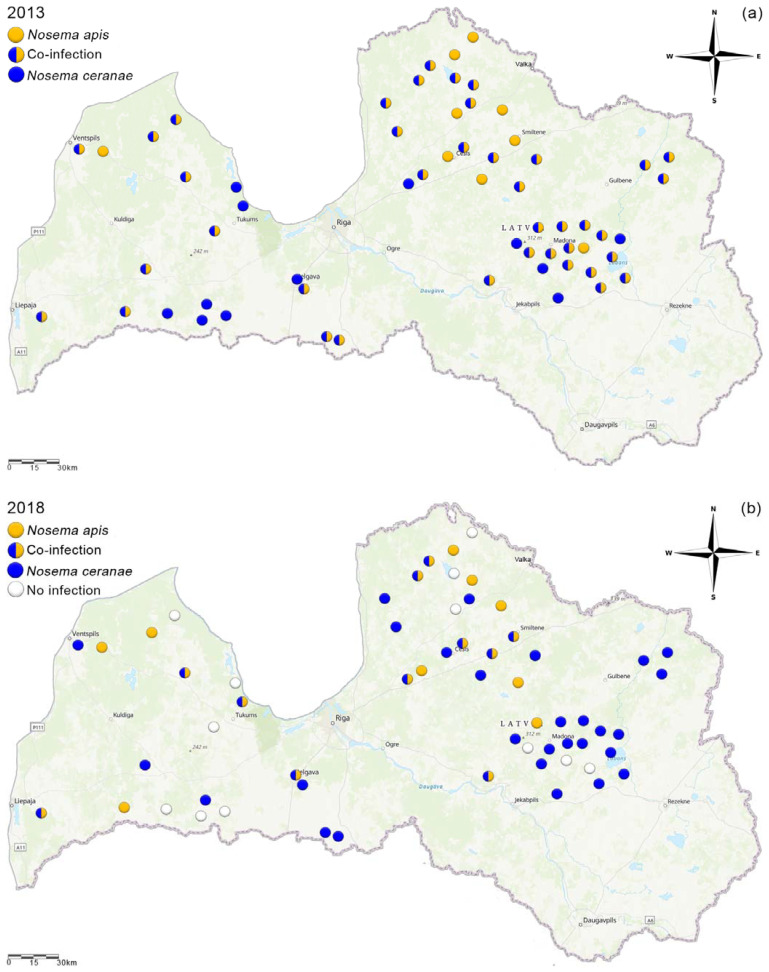
*Nosema* species prevalence in Latvia in 2013 (May) (**a**) and 2018 (May) (**b**) (ArcGis online).

A significant change in the distribution of *Nosema* species in Latvia was observed (χ^2^ = 35.71, *p* < 0.00001). In 2013, 65% of collected samples were co-infected (unpublished data from Latvian Beekeepers Association), 15% were infected with *N. apis*, and 20% were infected with *N. ceranae* (Figure 3a). However, in 2018, 47% of apiaries were infected with *N. ceranae*, 18% were infected with both species, and 15% were infected with *N. apis*; 20% of the apiaries in 2018 were those in which we could not find any *Nosema* (Figure 3b).

## 4. Discussion

Our study showed that both *Nosema* species causing nosemosis are present in Estonia and Latvia. The causative agents were found for both single infections and co-infections. Interestingly, the species composition varied greatly between the two neighboring countries at the time of sampling. *N. apis* was the most abundant species in Estonia, while *N. ceranae* was the most abundant species in Latvia. We observed similar spore counts for single-species infections of both species, whereas co-infected colonies had higher spore counts.

Since its first discovery in *A. mellifera* in Taiwan in 2005 [10], *N. ceranae* has become dominant in *Nosema*-infected apiaries. Over the last few decades, the original pathogenic species, *N. apis*, has been displaced by this more aggressive pathogen. Although for a long time, these two species were thought to be host species specific [13]. It is now clear that these species can exist either individually or together. Still, there is a question of whether a co-infection is more damaging to honey bees than a single infection. In cage studies where the spores of the two pathogens were fed to infection-free honey bees, both *Nosema* spp. were found to be virulent. This was especially true for *N. ceranae* and a mixture of *N. ceranae* and *N. apis*, as both groups of honey bees showed decreased longevity and viability [33,34]. This decrease in survival may result from the reduced defence mechanisms of the honey bees which makes the host more vulnerable to various external factors [35]. In addition, *N. ceranae* tends to be more aggressive during the rapid development phase of honey bee colonies, which in turn contributes to the increased number of spores [15]. In our study, the median number of spores per worker bee ranged from 1.6 to 14 million. A very similar result was shown by Odemer et al. [36]. Although they infected bees artificially and reported cross-infection of their *N. apis* honey bees. This could be explained by the fact that the artificial *Nosema* infection relies on viable spore material. Maybe the spores used for the *N. apis* infection were somehow of lower viability than in a natural setting and expressed therefore lesser spores in the infected honey bees. Additionally, at the end of the paper, they mention the fact that laboratory or semi-laboratory results depend on many factors which may affect the results. It is difficult to draw convincing conclusions about the correlation between high spore counts and colony losses [37] because in field and semi-field studies it is difficult to exclude the possible co-effects of other factors. Another question is whether an infection with these pathogens can lead to colony losses. Exposure to various stressors (pesticides, parasites, etc.) can significantly increase mortality. For example, exposure to various neonicotinoids (e.g., clothianidin and imidacloprid). However, here too, the results diverge. Alaux et al. [38] demonstrated that an interaction between *Nosema* spores and imidacloprid reduced the lifespan of honey bees and neonicotinoid exposure weakened colonies. Odemer et al. [36] demonstrated that by applying neonicotinoid clothianidin in field-relevant sublethal concentrations to free-flying colonies, the neonicotinoid clothianidin did not act synergistically either with *N. apis* or with *N. ceranae*. However, this neonicotinoid is considered to be very toxic to honey bees [39].

Estonia and Latvia are small neighboring countries with similar climates, thus climatic variation is an unlikely cause of the differences in species distribution. It is possible that *N. ceranae* is still increasing its range. This could be investigated by repeating the study after a shorter time and including random hives. A similar increase in the range of *N. ceranae* was also shown by Ostroverkhova et al. [40]; despite large climatic differences in the study region, they were not able to show a climate dependence in the relative spread of these two species. Pacini et al. [16] recorded infections with *N. apis* only in the subtropical regions of Argentina, whereas in the temperate regions, *N. apis* was detected only in co-infected colonies. Conversely, only *N. ceranae* was found in Saudi Arabia [41]. Finally, a study from Mexico showed that *N. apis* was dominant (87%) in an area with a warm climate [42].

Estonia seems to be one of the few countries in the world where *N. apis* is still individually prevalent. In a 4-year study carried out in Lithuania [22] which also looked at the distribution of these pathogens, their proportions were very similar, and neither species was dominant. This result is in contrast to our findings in Estonia and Latvia, where *N. apis* was prevalent in Estonia and *N. ceranae* was prevalent in Latvia. It has reported that *N. ceranae* has become increasingly dominant in Finland since 2006, whereas before that, *N. apis* was dominant [21]. It is possible that the geographical location of Estonia could explain the unique prevalence of *N. apis,* since honey bees cannot cross the Baltic Sea from Finland, the natural spread of honey bee pathogens could occur only from the south or east. However, *Nosema* can spread with the assistance of people, through the import of infected colonies, small nuclei of colonies or queens. In Estonia, imported queens are widely used. These queens come mainly from Romania and Italy, where *N. ceranae* is a common pathogen [43,44]. However, this does not explain why *N. apis* is more common in Estonia.

As there is no effective cure for *Nosema*, beekeepers need to use uncontaminated equipment in apiaries. The internal temperature of a honey bee colony is always 32–35 °C, which is a favorable temperature for the pathogen. A cold climate could somewhat aid in the fight against *Nosema*. This has led beekeepers to believe that cold storage of beekeeping equipment may kill the spores. However, studies have shown contrary and largely variable results. For example, Ozgor and Keskin [45] showed that *N. ceranae* is still infectious after 1 year at −20 °C. They also found that a milder cold temperature (4 °C) was even more conducive to spore survival. Finally, according to Fenoy et al. [46], there was no significant increase in spore mortality within a few hours or after a few months when the spores were exposed to a warm (35 °C) or very warm (60 °C) environment. This indicates that sterilization of beekeeping equipment after every usage in infected colonies is important to avoid spreading the spores from one apiary to another.

Our study samples were collected once in the spring when the age distribution of honey bee colonies exchange for younger bees. However, only one sampling date may create a situation where the *Nosema* prevalence may shift during the season and after overwintering. For future studies, we recommend at least three sampling times (spring, summer, autumn) to investigate the seasonality of the two pathogens in areas with colder climates. Moreover, to get an idea of the species distribution, similar samples should be taken over several years. It is still unclear whether the two pathogen species exhibit seasonality over a longer period. Based on previous studies, *N. apis* infection is present predominantly during the springtime. This is because in spring, only old bees that have overwintered in the hive are present, the queen is just beginning to lay eggs, and the laying intensity is low. Old bees are more susceptible to infection, and when nursing the hive, spores spread through the faecal-oral route [47] and reinfect the overwintered worker bees. The latter may also be true for *N. ceranae*. However, several studies have confirmed that it is difficult to find clear links between seasonality and species occurrence. Although spore counts are usually higher in the springtime, they can vary annually and depend on several other factors [19,26,35]. In our experiment, forager bees were collected, which may have affected the study results, because, according to Meana et al. [48], in-hive bees had fewer spore counts than foragers. In the future, in-hive bees should be examined to better describe the extent of the infection in the colony.

## 5. Conclusions

We conclude that the spread of *N. ceranae* may be lesser in regions with colder climates, but further research is needed. To clarify the threat of nosemosis to honey bees in various regions, we need to understand the co-effects of various stressors on infection severity, as well as how to protect honey bees so that their immune system can more effectively fight these internal pathogens. Additionally, from a long-term perspective, nation-wide and pan-European monitoring programs should cover the spread of *Nosema* more accurately and future research should focus on establishing such networks.

## Figures and Tables

**Table 1:** Primers used to detect *Nosema ceranae* and *Nosema apis*.

Name	Primer Sequence	Fragment Size (bp)	Specificity
Mn*Ceranae*-F	5′−CGTTAAAGTGTAGATAAGATGTT−3′	143	* N. ceranae *
Mn*Apis*-F	5′−GCATGTCTTTGACGTACTATG−3′	224	* N. apis *
Muniv-R	5′−GACTTAGTAGCCGTCTCTC−3′		

## Data Availability

Data sharing not applicable.
